# Stratifying Cardiovascular Risk in Patients With HCC Receiving Atezolizumab/Bevacizumab: A Comparison of ESC and CARDIOSOR Scores

**DOI:** 10.1111/liv.70909

**Published:** 2026-10-06

**Authors:** Bernardo Stefanini, Lorenza Rimassa, Massimo Iavarone, Giuseppe Cabibbo, Fabio Marra, Caterina Vivaldi, Andrea Dalbeni, Francesca Romana Ponziani, Andrea Palloni, Sara Lonardi, Lorenzo Lani, Piera Federico, Gianluca Svegliati‐Baroni, Tiziana Pressiani, Luca Ielasi, Chiara Mazzarelli, Stefania De Lorenzo, Rodolfo Sacco, Mariangela Bruccoleri, Ciro Celsa, Alessio Mastro, Gianluca Masi, Alessandra Auriemma, Leonardo Stella, Pierluigi Toniutto, Rosanna Villani, Chiara Deiana, Sara Ascari, Mariarosaria Marseglia, Alessandro Granito, Fabio Piscaglia, Francesco Tovoli, the ARTE Study Group, Maria Boe, Andrea De Sinno, Federico Ravaioli, Eleonora Alimenti, Lorenzo Canova, Anna Perna, Marco Vicardi, Leonardo A. Natola, Aldo Filice, Filippo Cattazzo, Federica Carlino, Giulia Grassetti, Lucia Cerrito, Francesco Santopaolo, Maria Pallozzi, Maurizio Pompili, Antonio Gasbarrini, Alessio Quartararo, Sofia Calascibetta, Federica Tosi, Arianna Toscano, Claudia Campani, Elisa Pellegrini

**Affiliations:** ^1^ Department of Medical and Surgical Sciences University of Bologna Bologna Italy; ^2^ Department of Biomedical Sciences Humanitas University Pieve Emanuele Milan Italy; ^3^ Hepatopancreatobiliary Oncology Unit, Humanitas Cancer Center IRCCS Humanitas Research Hospital Rozzano Milan Italy; ^4^ Division of Gastroenterology and Hepatology Foundation IRCCS Ca' Granda Ospedale Maggiore Policlinico Milan Italy; ^5^ Department of Pathophysiology and Transplantation, CRC ‘A. M. and A. Migliavacca’ Centre for Liver Disease University of Milan Milan Italy; ^6^ Gastroenterology and Hepatology Unit, Department of Health Promotion, Mother & Child Care, Internal Medicine & Medical Specialties University of Palermo Palermo Italy; ^7^ Internal Medicine and Liver Unit University Hospital Careggi, University of Florence Florence Italy; ^8^ Department of Experimental and Clinical Medicine University of Florence Florence Italy; ^9^ Unit of Medical Oncology 2 Azienda Ospedaliero‐Universitaria Pisana Pisa Italy; ^10^ Unit of General Medicine C, Medicine Department University of Verona and University and Hospital Trust (AOUI) of Verona Verona Italy; ^11^ Liver Unit, Medicine Department University of Verona and University and Hospital Trust (AOUI) of Verona Verona Italy; ^12^ Liver Unit, CEMAD Centro Malattie dell'Apparato Digerente, Medicina Interna e Gastroenterologia Fondazione Policlinico Universitario Gemelli IRCCS Rome Italy; ^13^ Dipartimento di Medicina e Chirurgia Traslazionale Università Cattolica del Sacro Cuore Rome Italy; ^14^ Medical Oncology IRCCS Azienda Ospedaliero‐Universitaria di Bologna Bologna Italy; ^15^ Oncology Unit 1 Veneto Institute of Oncology IOV—IRCCS Padua Italy; ^16^ Unit of Semeiotics, Liver and Alcohol‐Related Diseases IRCCS Azienda Ospedaliero‐Universitaria di Bologna Bologna Italy; ^17^ Oncology Unit Ospedale del Mare Napoli Italy; ^18^ Liver Injury and Transplant Unit Polytechnic University of Marche Ancona Italy; ^19^ Department of Internal Medicine Ospedale Degli Infermi di Faenza Faenza Italy; ^20^ Hepatology and Gastroenterology Unit ASST Grande Ospedale Metropolitano Niguarda Milan Italy; ^21^ Oncology Unit Azienda USL Bologna Bologna Italy; ^22^ Gastroenterology and Digestive Endoscopy Unit Foggia University Hospital Foggia Italy; ^23^ Department of Surgery and Cancer Imperial College London, Hammersmith Hospital London UK; ^24^ Section of Innovation Biomedicine‐Oncology Area, Department of Engineering for Innovation Medicine (DIMI) University of Verona and University and Hospital Trust (AOUI) of Verona Verona Italy; ^25^ Hepatology and Liver Transplantation Unit, Azienda Ospedaliero Universitaria University of Udine Udine Italy; ^26^ Liver Unit, Department of Medical and Surgical Sciences University of Foggia Foggia Italy; ^27^ Division of Internal Medicine, Hepatobiliary and Immunoallergic Diseases IRCCS Azienda Ospedaliero‐Universitaria di Bologna Bologna Italy

## Abstract

**Background & Aims:**

The management of comorbidities has become increasingly relevant in unresectable hepatocellular carcinoma (uHCC) as immunotherapy prolongs survival. Atezolizumab plus bevacizumab (AB) is a standard first‐line option, but bevacizumab carries a risk of cardiovascular toxicities. Data on cardiovascular risk stratification in patients with cirrhosis remain limited. Identifying patients at increased cardiovascular risk is therefore clinically important, particularly given the availability of bevacizumab‐free alternatives.

**Methods:**

We retrospectively analysed prospectively collected data from the multicenter Italian ARTE database, including patients with uHCC treated with first‐line AB between June 2022 and July 2025. Traditional major adverse cardiovascular events (tMACE) were defined as acute coronary syndromes, heart failure, cerebrovascular events, or peripheral ischemia, while an extended composite (eMACE) additionally included venous thromboembolism and immune‐related myocarditis. Cardiovascular risk was stratified using the European Society of Cardiology (ESC) Cardio‐Oncology classification and the CARDIOSOR score. Competing‐risk analyses were performed considering treatment discontinuation as a competing event.

**Results:**

This study included 538 patients (median age 69.8 years). During follow‐up, 20 patients (3.7%) experienced tMACE, and 30 (5.6%) experienced eMACE. Patients assigned to the very high‐risk category by ESC criteria and those with a high‐risk CARDIOSOR score had a significantly increased risk of tMACE and eMACE. Both scores demonstrated a moderate ability to stratify cardiovascular risk (Harrell's c‐index 0.68 for ESC and 0.59 for CARDIOSOR).

**Conclusions:**

Cardiovascular adverse events were uncommon overall, but their incidence was not negligible in patients in high‐risk subgroups. Risk scores may help identify vulnerable subgroups and support patient‐tailored treatment strategies, including consideration of bevacizumab‐free regimens in selected cases.

**Trial Registration:**

Multicenter ARTE database (NCT06806579)

## Key Points

- As survival improves in unresectable HCC, cardiovascular comorbidities are increasingly relevant to long‐term management.
- In our study, cardiovascular adverse events were uncommon overall with atezolizumab‐bevacizumab, but occurred more frequently in selected higher‐risk subgroups.
- Events occurred early during treatment and frequently led to bevacizumab interruption or permanent discontinuation.
- ESC and CARDIOSOR stratified cardiovascular risk, although their overall discriminative performance was moderate.
- Baseline cardiovascular risk assessment may support patient‐tailored treatment strategies, including consideration of bevacizumab‐free regimens in selected patients.

## Lay Summary

Atezolizumab plus bevacizumab is a standard treatment for advanced liver cancer, but one of its components can occasionally cause serious heart or circulatory events. Using data from more than 500 patients across Italy, we examined whether two established cardiovascular risk scores could predict who is most likely to experience these complications. Both scores worked moderately well and may help doctors identify higher‐risk patients for whom an alternative treatment could be considered.

## 1 Introduction

Immune checkpoint inhibitors (ICI) have substantially improved survival outcomes in patients with hepatocellular carcinoma (HCC), with a meaningful proportion of patients now achieving long‐term survival [1, 2, 3, 4, 5]. Currently, several first‐line immunotherapy‐based regimens are widely available: atezolizumab plus bevacizumab (AB), durvalumab plus tremelimumab (DT), and nivolumab plus ipilimumab. Camrelizumab plus rivoceranib also demonstrated an improvement in overall survival (OS) compared with tyrosine kinase inhibitors [3, 6].

In the absence of head‐to‐head comparative trials or validated predictive biomarkers, treatment selection in routine clinical practice is largely driven by patient‐related factors, including comorbidities. Cardiovascular risk factors are common in patients with HCC, a trend partly driven by the increasing burden of metabolic dysfunction–associated steatotic liver disease (MASLD), which is intrinsically associated with increased cardiovascular risk [7, 8, 9].

Bevacizumab has been associated with a spectrum of vascular and thrombotic toxicities [10], whereas ICI may cause immune‐mediated cardiovascular events, such as myocarditis, although infrequently. In patients with HCC treated with AB, clinical attention has traditionally focused on the bleeding risk [11]. Conversely, cardiovascular adverse events have received comparatively less attention, partly due to the historically limited life expectancy of these patients. However, in the current era of prolonged survival, cardiovascular toxicity may substantially affect treatment continuity, quality of life, and overall patient management.

A further challenge in this context is the lack of a universally accepted definition of major adverse cardiovascular events (MACE) in oncology and cardio‐oncology [12, 13, 14]. Traditional MACE (tMACE) definitions primarily include atherothrombotic events such as myocardial infarction, heart failure (both diastolic and systolic), and cerebrovascular events [15, 16]. However, other cardiovascular events, such as venous thromboembolism and immune‐mediated myocarditis, are increasingly recognised as clinically relevant in patients receiving both anti‐VEGF therapies and ICI. Accordingly, some studies have adopted broader composite endpoints that incorporate these events into an extended definition of MACE (eMACE) [17].

In parallel, different cardiovascular risk stratification tools have been developed to support clinical decision‐making in patients starting anticancer treatment, including the recommendations of the European Society of Cardiology (ESC) Cardio‐Oncology Group and the CARDIOSOR score [13, 14]. The ESC recommendations provide a general framework for baseline cardiovascular risk assessment across different cancer types and therapies, but have not been specifically validated in patients with HCC. Conversely, the CARDIOSOR score was originally developed in patients with HCC treated with sorafenib and has only recently been explored in patients receiving AB [17], without external validation. As a result, evidence supporting the applicability and comparative performance of these tools in contemporary populations with HCC remains limited.

In this context, a better characterisation of cardiovascular risk in patients with HCC treated with AB could ultimately support a more individualised approach to first‐line treatment selection, particularly when considering alternative immunotherapy‐based regimens.

## 2 Methods

### 2.1 Study Population

We conducted a retrospective analysis of prospectively collected data from the multicenter ARTE database (NCT06806579), which contains data from patients with unresectable HCC treated with immune‐based systemic treatments, starting in 2022. Major adverse cardiovascular events (MACE) were predefined within the registry protocol and systematically captured during follow‐up.

Data are entered at least biannually using a dedicated REDCap platform [18]. Data quality is ensured through periodic internal consistency checks performed at the data management centre of IRCCS Azienda Ospedaliero‐Universitaria di Bologna.

### 2.2 Patient Selection

We selected patients who received AB as first‐line systemic treatment for unresectable HCC between the start of data collection and the most recent database update, completed in July 2025.

### 2.3 Definition of ‘Traditional MACE’ and ‘Extended MACE’

For the primary analysis, we focused on tMACE, defined as the occurrence of acute coronary syndrome, heart failure, cerebrovascular events, or acute peripheral ischemia. These events were selected as the primary cardiovascular endpoint because they represent well‐established vascular complications potentially associated with anti‐VEGF therapy and are therefore directly relevant to treatment decision‐making in patients considered for bevacizumab‐containing regimens.

In addition, we explored an extended cardiovascular composite (eMACE), which included tMACE and: (1) venous thromboembolic events and (2) clinically relevant immune‐mediated myocarditis. Although characterised by distinct pathophysiological mechanisms, these events have been increasingly recognised as clinically meaningful cardiovascular events in patients receiving immunotherapy regimens. Analyses of eMACE were conducted as secondary and exploratory endpoints.

Cardiovascular events were prospectively recorded by the participating centers based on medical records. All events required specialist evaluation and were diagnosed according to established diagnostic criteria. Depending on the clinical presentation, the appropriate specialists were involved according to routine clinical practice (e.g., cardiologists for acute coronary syndromes, neurologists or internists for cerebrovascular events, and vascular surgeons or internists for peripheral ischemia).

### 2.4 ESC Cardio‐Oncology Score

The following items were systematically considered for every patient before starting AB as part of the standard clinical practice and in line with the ESC recommendations: age, previous history of cardiovascular disease (including but not limited to heart failure, history of angina, history of deep vein thrombosis or pulmonary embolism), generic cardiovascular risk factors (including hypertension, chronic kidney disease, diabetes, hyperlipidemia), previous or current cancer treatment, and lifestyle risk factors. Additionally, evaluations by a cardiologist and pertinent tests (including cardiac markers and imaging, when appropriate) were performed in patients with a history of cardiovascular events or multiple cardiovascular risk factors, as part of the usual clinical practice.

Risk categories were assigned using the ESC algorithm: patients with at least one very high‐risk factor were classified as very high risk; high risk included patients with at least one high‐risk factor or a cumulative score ≥ 5 from moderate factors; moderate risk corresponded to a cumulative score of 2–4; and low risk included patients without high‐ or very high‐risk factors and a total score < 2.

### 2.5 CARDIOSOR Score

The CARDIOSOR score was calculated as originally described [13]. Points were assigned for each of the following conditions: one point for arterial hypertension, diabetes mellitus, and dyslipidemia; two points for age ≥ 65 years; and three points for a history of ischemic heart disease, stroke, or peripheral vascular disease. Patients with a total score ≤ 4 were classified as low risk, whereas those with a score > 4 were classified as high risk.

### 2.6 Statistical Analysis

Continuous variables were summarised using the median and interquartile range (IQR), while categorical variables were reported as absolute counts and percentages.

Time to cardiovascular events was defined as the interval from the initiation of AB to the first cardiovascular event during treatment or within 30 days of treatment discontinuation. The 30‐day window was adopted to capture events plausibly related to recent treatment exposure.

Given the high mortality risk associated with advanced HCC, treatment discontinuation for any reason (including death or disease progression) prior to the occurrence of a cardiovascular event was considered a competing event. The cumulative incidence of tMACE and eMACE was estimated using competing‐risk methods. Fine–Grey subdistribution hazard models were used to evaluate the association between cardiovascular risk stratification tools and cardiovascular events. Subdistribution hazard ratios (sHRs) with 95% confidence intervals (CIs) were reported.

The discriminative performance of the ESC and CARDIOSOR risk stratification tools for predicting tMACE and eMACE was evaluated within the competing‐risk framework. Model performance was compared using the Akaike information criterion (AIC), the Bayesian information criterion (BIC), and Harrell's concordance index, adapted for competing‐risks data.

As an additional assessment of the robustness of the observed discriminative performance, bootstrap optimism correction was performed using Harrell's bootstrap procedure with 1000 resamples. For each resample, the model was refitted and the concordance index was calculated both in the bootstrap sample and in the original cohort. The mean difference between the two estimates was taken as the optimism estimate and subtracted from the apparent concordance index to obtain the optimism‐corrected concordance index.

Considering the expected low prevalence of tMACE and eMACE, risk categories were also evaluated dichotomously to enhance the stability of the estimates.

Survival analyses were conducted according to the Kaplan–Meier method; hazard ratios were calculated using Cox proportional hazard models for baseline variables while a time‐dependent analysis was used to assess the impact of adverse events occurring during treatment.

All statistical tests were two‐sided, and *p*‐values < 0.05 were considered statistically significant. Statistical analyses were performed using STATA 19/SE.

### 2.7 Ethics

The study protocol was approved by the Ethics Committee of the coordinating centre (IRCCS Azienda Ospedaliero‐Universitaria di Bologna; study code: 811.2022.Oss.AOUBo). All patients provided written informed consent in accordance with the Ethics Committee's recommendations. The study was conducted in accordance with the ethical guidelines of the 1975 Declaration of Helsinki.

## 3 Results

Between the start of the ARTE database and its latest update, 638 patients received first‐line systemic therapy for HCC. Of these, 100 received regimens other than AB and were excluded. The final study population consisted of 538 patients.

### 3.1 Study Population

Baseline characteristics of the study population are summarised in Table 1. Most patients were males, with a median age of 69.8 years, and 78.1% had a clinical diagnosis of liver cirrhosis at treatment initiation. Cardiovascular risk factors were common: more than half of the cohort had arterial hypertension, 20% had obesity, and 7.6% had a prior history of chronic coronary artery disease. The median follow‐up was 22.4 months (95% CI 20.9–24.0).

**TABLE 1:** Baseline characteristics of the whole cohort (*n* = 538) Variables are expressed as frequencies and percentages.

Baseline characteristics	Total cohort *n* = 538 (%)
Age (years), median (IQR)	69.8 (61.4–76.8)
< 65 years	189 (35.1)
65–74 years	179 (33.3)
≥ 75 years	170 (31.6)
Male	438 (81.4)
Cirrhosis	455 (84.6)
Child Pugh A	484 (90.0)
ALBI grade 1	268 (49.8)
Macrovascular invasion	195 (36.2)
Extrahepatic spread	203 (37.7)
ECOG‐PS 1	165 (30.7)
AFP > 400 ng/mL	151 (28.1)
*Aetiology attributed* ^a^	
HBV	97 (18.0)
HCV	247 (45.9)
MASLD	143 (26.6)
ALD	101 (18.8)
Others (non‐viral)	31 (5.8)
*Cardiovascular risk factors*	
Arterial hypertension	303 (56.3)
Type 2 diabetes mellitus	177 (34.7)
Dyslipidaemia	126 (24.9)
Peripheral vascular disease	65 (12.1)
Chronic kidney disease	26 (5.1)
Current Smokers	19 (3.5)
Obesity (BMI > 30)	100 (18.4)
CAD	41 (7.6)
*Lab values*	
Bilirubin (mg/dL), median (IQR)	0.86 (0.57–1.27)
Albumin (g/dl), median (IQR)	3.9 (3.6–4.2)
INR, median (IQR)	1.1 (1.02–1.2)

Abbreviations: ALBI, albumin‐bilirubin; ALD, alcohol liver disease; BMI, Body Mass Index; CAD, coronary artery disease; ECOG‐PS, Eastern Cooperative Oncology Group‐Performance Status; INR, International Normalised Ratio; IQR, Interquartile Range; MASLD, Metabolic Dysfunction‐Associated Steatotic Liver Disease.

^a^ Multiple etiologies were reported in 105 patients.

### 3.2 Cardiovascular Risk Stratification

According to the CARDIOSOR score, most patients (*n* = 449, 83.9%) fell into the low‐risk category. Similarly, a significant proportion of patients were classified in the low‐ or moderate‐risk category of the ESC Cardio‐Oncology algorithm (Figure 1). The baseline characteristics of the patients, according to their risk classes, are reported in Tables S1 and S2.

**FIGURE 1:**
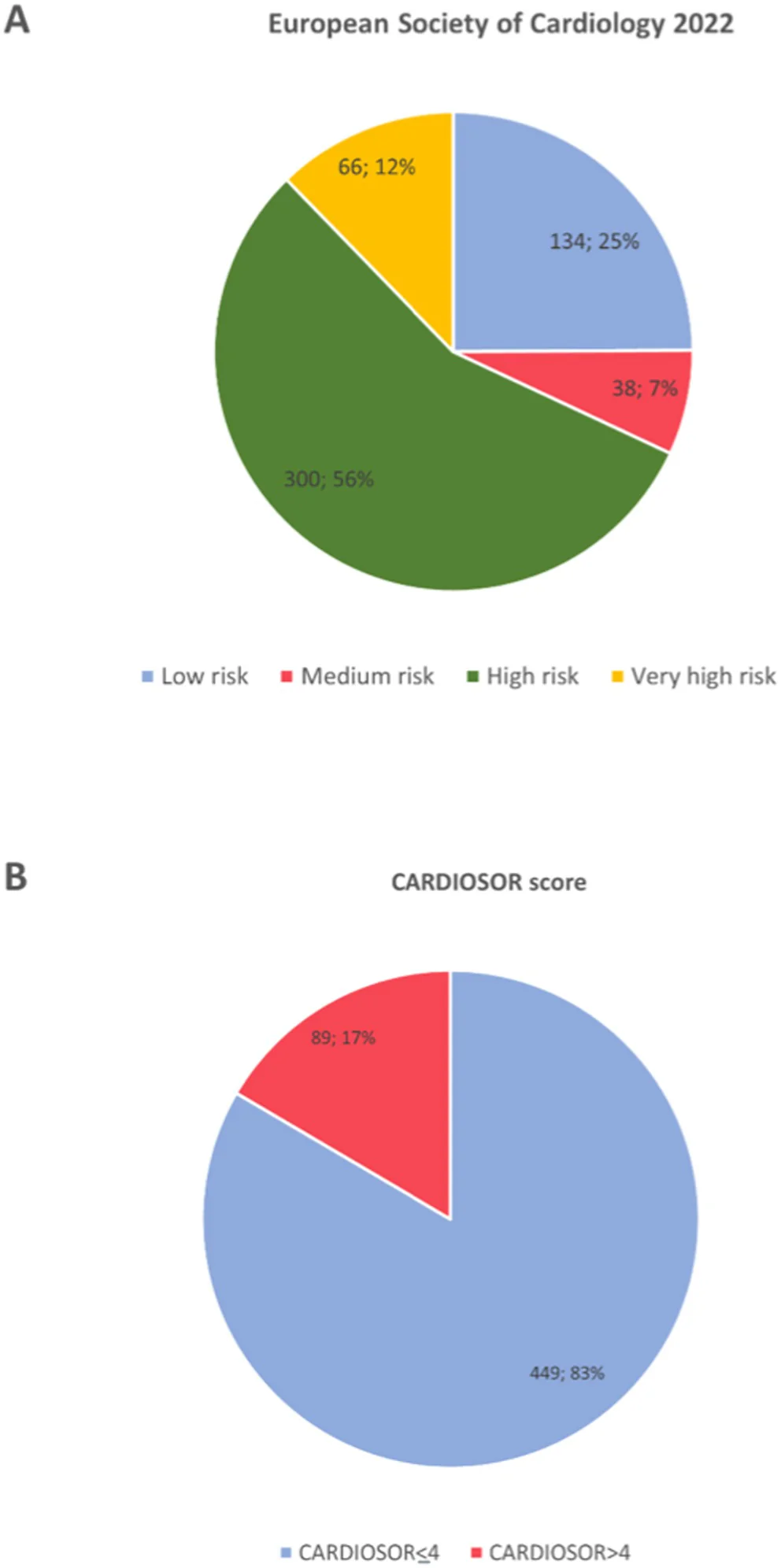
European Society of Cardiology (ESC) 2022 (A) and CARDIOSOR (B) classification in the study cohort (*n* = 538).

### 3.3 Traditional MACE

During the follow‐up, 20 (3.7%) patients experienced a tMACE, including cerebrovascular accidents (*n* = 8), acute coronary syndrome (*n* = 7), peripheral limb ischaemia (*n* = 3), and heart failure (*n* = 2). The median time from initiation of AB to the occurrence of tMACE was 4.8 months (95% CI 2.4–7.2 months).

Five events were fatal. In the remaining cases, bevacizumab was permanently discontinued in ten patients and temporarily interrupted in five patients. In patients who resumed bevacizumab, the median duration of treatment interruption was 3.4 months.

No individual cardiovascular risk factor was significantly associated with tMACE, except for chronic kidney disease (Table S3).

### 3.4 Extended MACE

Overall, 30 patients (5.6%) experienced an eMACE during follow‐up, with a median time to event of 4.0 months (95% CI, 2.5–5.5). In addition to the 20 tMACE events, two patients (0.3%) developed immune‐mediated myocarditis, and eight patients (1.5%) experienced venous thromboembolic events, including 2 fatal events. Both components of AB were discontinued in the event of myocarditis. In patients with thromboembolic events, bevacizumab was discontinued in five cases and interrupted in three.

No individual cardiovascular risk factor was associated with the occurrence of eMACE (Table S4).

### 3.5 Discriminative Abilities of ESC and CARDIOSOR

Both the ESC Cardio‐Oncology and the CARDIOSOR scores stratified the risk of tMACE and eMACE in the study population (Figure 2).

**FIGURE 2:**
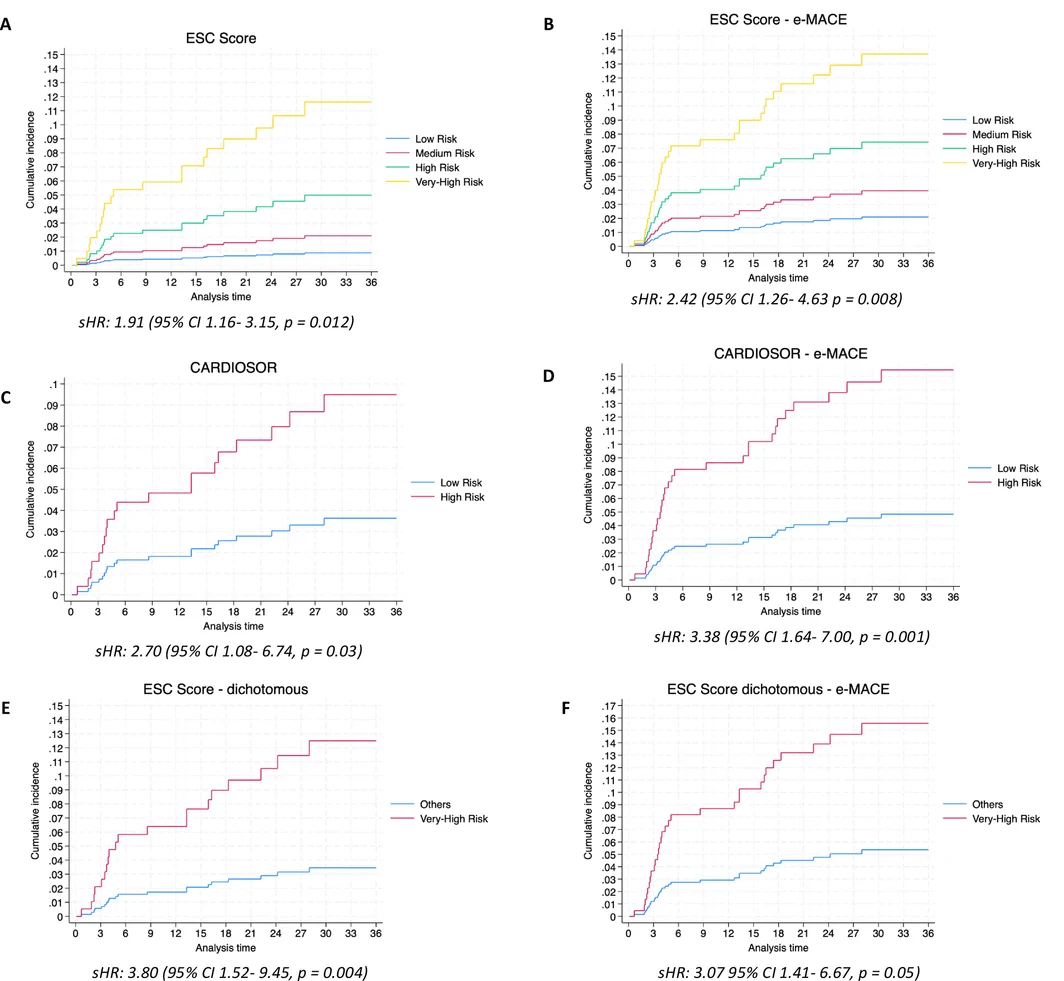
Risk of traditional (tMACE) and extended major adverse cardiovascular events (eMACE) according to the European Society for Cardiology (ESC) 2022 and CARDIOSOR scores. Stratification was performed according to the 4‐tier ESC score evaluating tMACE (A) and eMACE (B). Similar analyses were performed according to the CARDIOSOR score (C and D, respectivily) and the dichotomized ESC score (E and F, respectively).

When the ESC risk categories were analysed, patients in the very high‐risk stratum (*n* = 66, 12.3%) had a higher cumulative incidence of both tMACE and eMACE than those in the reference category (Figure 2). In particular, the cumulative incidence of events in the very‐high‐risk group was 11.8% for tMACE and 13.6% for eMACE. In a dichotomised analysis (very high‐risk vs. all other categories), the median sHR was 3.80 (95% CI, 1.52–9.45; *p* = 0.004) (Table 2).

**TABLE 2:** Cumulative risk of traditional major cardiovascular adverse events (tMACE) according to the competing risk analysis.

Risk score	Patients (%)	Events (%)	1‐year cumulative incidence (%, 95% CI)	2‐year cumulative incidence (%, 95% CI)	sHR (95% CI)	*p*
ESC 2022						
Low	134 (24.9)	1 (0.8)	0	0.7 (0.1–7.6)	Reference	
Moderate	38 (7.1)	1 (2.6)	1.9 (0.3–18.4)	1.9 (0.3–18.4)	3.67 (0.23–58.46)	0.357
High	300 (55.8)	11 (3.7)	2.1 (0.9–4.6)	4.1 (2.0–6.9)	4.99 (0.65–38.30)	0.122
Very high	66 (12.3)	7 (10.6)	7.7 (3.2–17.7)	9.9 (4.5–21.3)	14.18 (1.76–114.27)	**0.013**
CARDIOSOR						
Low	449 (83.4)	13 (2.9)	1.6 (0.8–3.4)	3.1 (1.8–5.5)	Reference	
High	89 (16.5)	7 (7.9)	5.8 (2.4–13.6)	8.3 (3.4–17.1)	2.70 (1.08–6.74)	**0.033**
ESC 2022 (dichotomised)						
Very high	66 (12.3)	7 (10.6)	7.7 (3.2–17.7)	9.9 (4.5–21.3)	3.80 (1.52–9.45)	**0.004**
Other	472 (87.7)	13 (2.7)	1.5 (0.7–3.2)	3.2 (1.7–5.2)	Reference	

*Note:* Statistaclly significant *p* values were highlighted using “bold”.

Abbreviations: CI, confidence interval; ESC, European Society of Cardiology; sHR, subdistribution hazard ratio.

According to the CARDIOSOR score, patients in the high‐risk group had a higher incidence of tMACE than those in the low‐risk group, with a sHR of 2.70 (95% CI, 1.08–6.74; *p* = 0.03). A similar association was observed when eMACE were considered, with an sHR of 3.38 (95% CI, 1.64–7.00; *p* = 0.001) (Figure 2B). In detail, patients classified as high risk had cumulative incidences of 7.9% for tMACE and 13.5% for eMACE.

Both the AIC and BIC showed comparable model fit, with values of 235 and 239 for the ESC risk model and 241 and 245 for the CARDIOSOR score, respectively. The ESC Cardio‐Oncology risk stratification achieved a Harrell's concordance index of 0.68 (95% CI, 0.58–0.78), compared with 0.59 (95% CI, 0.47–0.71) for the CARDIOSOR score (Table 3).

**TABLE 3:** Accuracy of the European Society of Cardiology (ESC‐2022) score compared to the CARDIOSOR score to predict the occurrence of traditional major adverse cardiovascular events (tMACE).

Prognostic score	Harrell's c‐index apparent (95% CI)	*p* (Harrell)	Wolbers c‐index (95% CI)
ESC‐2022			
4 classes	0.68 (0.58–0.78)	Reference	0.65 (0.62–0.70)
2 classes	0.61 (0.49–0.73)	0.006	0.59 (0.52–0.66)
CARDIOSOR			
2 classes	0.59 (0.47–0.71)	0.001	0.57 (0.52–0.62)

*Note:* Accuracy is calculated according to tMACE‐free‐specific survival (Harrell) and competing‐risk survival (Wolbers). *p* values refer to comparison of Harrell's c‐statistics between the ESC‐2022 four‐classes model and the other risk scores.

Abbreviations: CI, confidence interval; ESC, European Society of Cardiology.

After bootstrap‐based optimism correction, concordance indices were only minimally changed, with optimism‐corrected values of 0.65 for the four‐category ESC stratification, 0.61 for the dichotomised ESC classification, and 0.58 for the CARDIOSOR score.

### 3.6 Prognostic Role of Cardiovascular Risk and MACE

Two hundred sixty‐two patients were alive at the time of data censoring, while 276 had died during the observation period. The median OS was 19.7 months (95% CI 17.1–22.3). We found no difference in OS when the population was stratified according to CARDIOSOR score. In detail, patients with a baseline high risk reached a median OS of 20.4 months (95% CI 12.5–28.3) compared to 19.5 months (95% CI 16.7–22.4) for patients with a low baseline risk score (*p* = 0.485).

The median OS was significantly longer in the ESC low‐risk category (26.1 vs. 19.7 months, *p* = 0.020), while survival curves largely overlapped across the remaining categories.

According to the time‐dependent Cox regression analysis, the occurrence of tMACE was associated with an increased risk of death (HR 8.08, 95% CI 5.59–11.66, *p* < 0.001). When considering eMACE, the results were similar (HR 8.52, 95% CI 5.88–12.34, *p* < 0.001).

## 4 Discussion

The availability of multiple immunotherapy‐based regimens for HCC has opened a door to patient‐tailored treatment strategies, in which clinical characteristics and comorbidities increasingly inform therapeutic decision‐making. In the absence of head‐to‐head comparative trials or validated predictive biomarkers, treatment selection between bevacizumab‐containing and bevacizumab‐free regimens is largely guided by the individual patient profile. In this context, cardiovascular comorbidities represent a particularly relevant consideration, given both their high prevalence in contemporary populations with HCC and the cardiovascular toxicity profile associated with anti‐VEGF therapy.

In this study, we provide real‐world evidence from a large multicenter cohort of patients with unresectable HCC treated with AB. The prospective data collection and broad inclusion criteria ensure a representation of routine clinical practice. An additional strength of our analysis is the use of a competing‐risk framework, which is particularly appropriate in patients with unresectable HCC, where treatment discontinuation and non‐cardiovascular events are frequent and may preclude the observation of cardiovascular outcomes.

The first relevant finding of our study is the cumulative incidence of tMACE and eMACE. The incidence of tMACE observed in our cohort is consistent with that reported in most previous real‐world studies evaluating systemic therapies in HCC [19]. Conversely, the incidence of eMACE was lower than that described in a recent study by Fortuny et al. [17]. This difference is likely explained by heterogeneity in endpoint definitions. In particular, asymptomatic cardiac biomarker elevations were included in the eMACE in that study, whereas our analysis included exclusively symptomatic myocarditis. Overall, our findings suggest a favourable cardiovascular safety profile of AB, while confirming that clinically relevant cardiovascular events, although infrequent, are not negligible.

The clinical impact of these events extends beyond their incidence. In fact, even when they were non‐fatal, cardiovascular adverse events were frequently associated with a permanent discontinuation or a prolonged interruption of bevacizumab. Notably, cardiovascular events typically occurred early after treatment initiation, an additional negative aspect of these events considering the growing proportion of long‐term survivors reported with immune‐based combinations. This observation is particularly relevant in the current therapeutic era in which non‐oncological events and treatment‐limiting toxicities may increasingly influence prognosis [4, 20].

These considerations underscore the need to identify patients at increased risk of cardiovascular complications. This aim represented the second major focus of our study, which compared the performances of the ESC and CARDIOSOR stratification tools. We found that both tools stratified cardiovascular risk and identified subgroups of patients with a higher incidence of tMACE and eMACE. Among the evaluated models, the four‐tier ESC classification showed the potential for greater discriminative ability, although the number of observed events limited our estimates. With respect to CARDIOSOR, our findings are concordant with previous reports from the sorafenib era [13] and offer an independent confirmation in a cohort of patients treated with AB. To the best of our knowledge, this is the first study to directly compare ESC and CARDIOSOR risk stratification tools in this clinical setting.

From a clinical perspective, cardiovascular risk stratification may contribute to more informed therapeutic decisions. In patients with a cardiovascular risk profile consistent with the highest risk category, a comprehensive cardiology evaluation and an optimisation of cardiovascular risk factors may be warranted prior to treatment initiation. In selected cases, the availability of effective bevacizumab‐free immunotherapy regimens may also support consideration of alternative treatment strategies. Importantly, such decisions should be individualised and integrated within a multidisciplinary framework rather than driven solely by risk scores.

Several limitations of this study should be acknowledged. First, the number of cardiovascular events was limited, reflecting the relatively low incidence of these outcomes. The relatively modest number of events may have reduced the precision of risk estimates and discriminative metrics, as reflected by the wide confidence intervals observed for selected categories. It may also have limited the ability to detect weaker associations between individual baseline variables and cardiovascular outcomes. Nevertheless, the observed associations between the predefined risk categories and cardiovascular outcomes were consistent across the primary and extended endpoints. Second, the absence of a control group treated with bevacizumab‐free regimens precludes causal attribution of cardiovascular events specifically to AB. However, because these regimens have only recently entered routine clinical practice, adequately powered patient cohorts for comparative studies are not yet available. Future comparative analyses will also require propensity score–based methods, given the expected prescription bias toward bevacizumab‐free treatments in patients with relevant cardiovascular comorbidities, even more stressing the need for very large study cohorts.

In conclusion, this study confirmed that cardiovascular adverse events represent a clinically relevant risk in patients with HCC. Established cardiovascular stratification tools can help identify subgroups of patients at increased risk. Although the discriminative ability of both scores was moderate, they consistently identified patient subgroups with substantially different absolute risks of cardiovascular events. This feature may be particularly relevant when treatment decisions involve the choice between bevacizumab‐containing and bevacizumab‐free regimens. These findings support integrating cardiovascular risk assessment into patient‐tailored treatment strategies and highlight the need for further studies to refine risk prediction and optimise therapeutic selection.

## Author Contributions

Bernardo Stefanini, Lorenza Rimassa and Francesco Tovoli conceived and designed the study. Bernardo Stefanini and Francesco Tovoli performed the statistical analysis. Bernardo Stefanini wrote the first draft. Lorenza Rimassa, Francesco Tovoli and Fabio Piscaglia supervised this research and provided overall leadership. All the authors contributed to the data acquisition, critically revised the manuscript and gave positive approval to the final version of the manuscript.

## Funding

The authors have nothing to report.

## Conflicts of Interest

Lorenza Rimassa received consulting fees from AbbVie, AstraZeneca, Basilea, Bayer, BMS, Boehringer Ingelheim, Boston Scientific, Eisai, Elevar Therapeutics, Exelixis, Genenta, Guerbet, Hengrui, Incyte, Ipsen, Jazz Pharmaceuticals, MSD, Nerviano Medical Sciences, Roche, Servier, Taiho Oncology, Zymeworks; lecture fees from AstraZeneca, Bayer, Biologix, BMS, Eisai, Guerbet, Incyte, Ipsen, Roche, Servier; research funding (to institution) from AbbVie, AstraZeneca, BeiGene, BMS, Exelixis, Fibrogen, Incyte, Ipsen, Jazz Pharmaceuticals, MSD, Nerviano Medical Sciences, Roche, Servier, Taiho Oncology, TransThera Sciences, Zymeworks; travel expenses from AstraZeneca, Servier. Giuseppe Cabibbo has served on an advisory board and received speaker fees for Bayer, Eisai, Ipsen, AstraZeneca, MSD, Roche, and Gilead. Massimo Iavarone received consulting fees from Roche, Roche Diagnostics, EISAI, MSD, Astra Zeneca; lecture fees from Roche, Astra Zeneca, IPSEN, EISAI, MSD, Gilead; travel fees from Roche; research funding (institution) from Gilead, Roche, Astra Zeneca, EISAI. Ciro Celsa received speaker fees for AstraZeneca, EISAI, MSD, Ipsen, and received grants from Roche. Francesca Romana Ponziani received speaker fees, advisory board fees and travel grants from Bayer, MSD, Roche, Eisai, Ipsen, Astra‐Zeneca, Gilead, Abbvie. Fabio Piscaglia has served on advisory boards for Astrazeneca, EISAI, Exact Sciences, MSD, Roche, and Siemens Healthineers; speeches at symposia for Astrazeneca, Bayer, Bracco, ESAOTE, EISAI, GE, IPSEN, MSD, Roche, and Samsung; and is a consultant for Bracco and Nerviano. Tiziana Pressiani received/reports consulting fees from Bayer, Ipsen and Astra Zeneca; institutional research funding from Roche, Bayer, AstraZeneca.; support for congress attendance from Roche. Sara Lonardi reports research funding from Amgen, Astellas, AstraZeneca, Bayer, Bristol Myers Squibb, Daiichi Sankyo, Hutchinson, Incyte, Merck Serono, Mirati, MSD, Pfizer, Roche, and Servier; consulting fees from Amgen, AstraZeneca, Bristol Myers Squibb, Daiichi Sankyo, Incyte, Lilly, Merck Serono, MSD, and Servier; and payment/honoraria for lectures/presentations from Amgen, Bristol Myers Squibb, Incyte, GSK, Lilly, Merck Serono, MSD, Pierre‐Fabre, Roche, and Servier. Andrea Dalbeni received/reports consulting fees from EISAI, Roche, AstraZeneca, Lilly, Novartis; support for congress attendance from Roche, EISAI, MSD, AstraZeneca, Bohering. Francesco Tovoli received consulting fees from Roche, Astrazeneca, Eisai, BMS. All the other authors do not report any conflict of interest.

## Supporting information


**Table S1:** Baseline characteristics of the patients across the different risk classes of the European Society of Cardiology score.
**Table S2:** Baseline characteristics of the patients across the different risk classes of the CARDIOSOR score.
**Table S3:** Univariate analysis of baseline characteristics associated with the development of traditional major cardiovascular adverse events (tMACE) (*n* = 20) according to competing risk regression.
**Table S4:** Univariate analysis of baseline characteristics associated with the development of extended major cardiovascular adverse events (eMACE) (*n* = 30) according to competing risk regression.

## Data Availability

The data that support the findings of this study are available from the corresponding author upon reasonable request. The data are not publicly available due to privacy and ethical restrictions.
