# Supplementary material for: Stratifying Cardiovascular Risk in Patients With HCC Receiving Atezolizumab/Bevacizumab: A Comparison of ESC and CARDIOSOR Scores
Source: Liver Int. 2026 Oct 6;46(11):e70909. doi: 10.1111/liv.70909 (PMC13643190; doi:10.1111/liv.70909)
Supplement: Supplementary file 1 [file LIV-46-0-s001.docx]

Supplementary Table 1. Baseline characteristics of the patients across the different risk classes of the European Society of Cardiology score.

| **Baseline characteristics**  **Whole cohort (n=538)** | **ESC low risk class**  **(n= 134, 24.9%)** | **ESC moderate risk class**  **(n= 38, 7.0%)** | **ESC high risk class**  **(n= 300, 55.8%)** | **ESC very high risk class**  **(n= 66, 12.3%)** |
| --- | --- | --- | --- | --- |
| Age (years), median (IQR) | 60.2 (54.3-65.3) | 67.0 (63.4-71.5) | 74.4 (65.9-78.8) | 73.6 (66.1-78.2) |
| Male | 109 (81.3) | 34 (89.5) | 232 (77.3) | 63 (95.5) |
| Cirrhosis | 127 (76.1) | 30 (78.9) | 219 (73.0) | 53 (80.3) |
| Child Pugh A | 118 (88.1) | 34 (89.5) | 271 (90.3) | 61 (92.4) |
| ALBI grade 1 | 64 (47.8) | 18 (47.4) | 150 (50.0) | 36 (54.5) |
| Macrovascular invasion | 54 (40.3) | 16 (42.1) | 99 (33.0) | 26 (39.4) |
| Extrahepatic spread | 60 (44.8) | 10 (26.3) | 107 (35.7) | 26 (39.4) |
| ECOG-PS 1 | 38 (28.4) | 11 (28.9) | 93 (31.0) | 23 (34.8) |
| AFP > 400 ng/ml | 37 (27.6) | 14 (36.8) | 87 (29.0) | 13 (19.7) |
| **Cardiovascular Risk factors (%)** |  |  |  |  |
| Arterial hypertension | 0 (0.0) | 0 (0.0) | 252 (84.0) | 51 (77.3) |
| Type 2 diabetes mellitus | 9 (6.7) | 28 (73.7) | 109 (36.3) | 31 (47.0) |
| Dyslipidaemia | 3 (2.2) | 18 (47.4) | 73 (24.3) | 32 (48.5) |
| Peripheral vascular disease | 0 (0.0) | 1 (2.6) | 0 (0.0) | 24 (36.4) |
| Chronic kidney disease | 1 (0.7) | 2 (5.3) | 19 (6.3) | 5 (7.6) |
| Current Smokers | 4 (3.0) | 1 (2.6) | 11 (3.7) | 3 (4.5) |
| Obesity (BMI>30) | 4 (3.0) | 16 (42.1) | 62 (20.7) | 18 (27.3) |
| CAD | 0 (0) | 0 (0) | 0 (0) | 41 (62.1) |
| **Lab values** |  |  |  |  |
| Bilirubin (mg/dL), median (IQR) | 0.86 (0.62-1.24) | 0.98 (0.74-1.42) | 0.86 (0.52-1.29) | 0.80 (0.54-1.17) |
| Albumin (g/dl), median (IQR) | 3.9 (3.5-4.2) | 3.8 (3.2-4.3) | 3.9 (3.6-4.2) | 4.0 (3.7-4.3) |
| INR, median (IQR) | 1.10 (1.04-1.21) | 1.10 (1.00-1.20) | 1.10 (1.02-1.20) | 1.11 (1.01-1.20) |

Abbreviations: ALBI (albumin-bilirubin); ECOG-PS (Eastern Cooperative Oncology Group-Performance Status); AFP (Alpha-fetoprotein); CAD (chronic coronary artery disease); IQR (Interquartile Range); BMI (Body Mass Index), ESC (European Society of Cardiology).

Supplementary Table 2. Baseline characteristics of the patients across the different risk classes of the CARDIOSOR score.

| **Baseline characteristics**  **Whole cohort (n=538)** | **Cardiosor low risk**  **(n= 449, 83.5%)** | **Cardiosor high risk**  **(n=89, 16.5%)** |
| --- | --- | --- |
| Age (years) | 67.7 (59.9-76.0) | 74.9 (70.3-79.0) |
| Male | 357 (79.5) | 81 (91.0) |
| Cirrhosis | 337 (75.1) | 67 (75.3) |
| Child Pugh A | 401 (89.3) | 83 (93.3) |
| ALBI grade 1 | 219 (48.8) | 49 (55.5) |
| Macrovascular invasion | 164 (36.5) | 31 (34.8) |
| Extrahepatic spread | 169 (37.6) | 34 (38.2) |
| ECOG-PS 1 | 133 (29.6) | 32 (36.0) |
| AFP > 400 ng/ml | 134 (29.8) | 17 (19.1) |
| **Cardiovascular Risk factors** |  |  |
| Arterial hypertension | 225 (50.1) | 78 (87.7) |
| Type 2 diabetes mellitus | 116 (25.8) | 61 (68.5) |
| Dyslipidaemia | 64 (14.3) | 62 (69.7) |
| Peripheral vascular disease | 5 (1.1) | 20 (22.5) |
| Chronic kidney disease | 15 (3.3) | 11 (12.4) |
| Current Smokers | 16 (3.6) | 3 (3.4) |
| Obesity (BMI>30) | 73 (16.3) | 27 (30.3) |
| CAD | 3 (0.7) | 38 (42.7) |
| **Lab values** |  |  |
| Bilirubin (mg/dL), median (IQR) | 0.88 (0.58-1.30) | 0.79 (0.53-1.15) |
| Albumin (g/dl), median (IQR) | 3.9 (3.6-4.2) | 3.9 (3.7-4.3) |
| INR, median (IQR) | 1.10 (1.03-1.20) | 1.10 (1.02-1.20) |

Abbreviations: ALBI (albumin-bilirubin); ECOG-PS (Eastern Cooperative Oncology Group-Performance Status); AFP (Alpha-fetoprotein); CAD (chronic coronary artery disease), IQR (Interquartile Range); BMI (Body Mass Index).

Supplementary Table 3. Univariate analysis of baseline characteristics associated with the development of traditional major cardiovascular adverse events (tMACE) (n=20) according to competing risk regression.

| **Variable** | **sHR** | **95% CI** | **P** |
| --- | --- | --- | --- |
| Age (>65years) | 3.11 | 0.92-10.53 | 0.068 |
| Sex (Male) | 1.11 | 0.37-3.29 | 0.855 |
| Hypertension | 1.38 | 0.55-3.45 | 0.491 |
| Obesity (BMI>30) | 1.33 | 0.45-3.98 | 0.604 |
| Chronic kidney disease | 3.64 | 1.07-12.46 | ***0.039*** |
| Ischemic Heart Disease | 2.01 | 0.60-6.78 | 0.261 |
| Dyslipidemia | 2.01 | 0.83-4.90 | 0.124 |
| Diabetes | 0.84 | 0.32-2.18 | 0.720 |
| Peripheral arterial occlusive disease | 3.66 | 0.51-26.46 | 0.199 |
| CP class B | 1.03 | 0.24-4.45 | 0.971 |

Abbreviations: CP (Child-Pugh); BMI (Body Mass Index); sHR (sub-distribution hazard ratio).

Supplementary Table 4. Univariate analysis of baseline characteristics associated with the development of extended major cardiovascular adverse events (eMACE) (n=30) according to competing risk regression.

| **Variable** | **sHR** | **95% CI** | **P** |
| --- | --- | --- | --- |
| Age (>65years) | 3.11 | 0.92-10.53 | 0.068 |
| Sex (Male) | 1.11 | 0.37-3.29 | 0.855 |
| Hypertension | 1.29 | 0.57-2.93 | 0.545 |
| Obesity (BMI>30) | 1.58 | 0.65-3.79 | 0.306 |
| Chronic Kidney Disease | 2.02 | 0.86-7.27 | 0.089 |
| Ischemic Heart Disease | 1.65 | 0.31-8.87 | 0.560 |
| Dyslipidemia | 1.46 | 0.65-3.29 | 0.361 |
| Diabetes | 0.60 | 0.26-1.39 | 0.235 |
| Peripheral arterial occlusive disease | 1.03 | 0.23-4.54 | 0.972 |
| CP class B | 0.97 | 0.22-4.19 | 0.967 |

Abbreviations: CP (Child-Pugh); BMI (Body Mass Index); sHR (sub-distribution hazard ratio); eMACE: extended major adverse cardiovascular events.
